# Expression of GnRH, Kisspeptin, and Their Specific Receptors in the Ovary and Uterus in Deslorelin-Treated Late-Prepubertal Bitches †

**DOI:** 10.3390/vetsci11120591

**Published:** 2024-11-25

**Authors:** Muhammet Ali Karadağ, Aykut Gram, Sabine Schäfer-Somi, Selim Aslan, Duygu Kaya

**Affiliations:** 1 Department of Obstetrics and Gynecology, Faculty of Veterinary Medicine, Kafkas University, Kars 36100, Türkiye; m.ali.karadag@kafkas.edu.tr; 2 Department of Histology and Embryology, Faculty of Veterinary Medicine, Erciyes University, Kayseri 38039, Türkiye; aykutgram@erciyes.edu.tr; 3 Clinical Center for Reproduction, Vetmeduni, 1210 Vienna, Austria; sabine.schaefer@vetmeduni.ac.at; 4 Department of Obstetrics and Gynecology, Faculty of Veterinary Medicine, Near East University, Nicosia 99138, Cyprus; selim.aslan@neu.edu.tr; 5 Department of Obstetrics and Gynecology, Faculty of Veterinary Medicine, Dokuz Eylül University, İzmir 35890, Türkiye

**Keywords:** deslorelin, prepubertal bitches, GnRH/GnRHR, KISS1/KISS1R

## Abstract

In this study, the expression and localization of gonadotropin-releasing hormone (GnRH1) and kisspeptin (KISS1) and their specific receptors in canine ovarian and uterine tissues were investigated after the application of deslorelin acetate (Suprelorin^®^, 4.7 mg, Virbac, France) in the late prepubertal period. We hypothesized that prolonged treatment of prepubertal dogs with deslorelin would alter the expression of GnRH and kisspeptin genes in the uterus and ovaries. Ovarian and uterine samples of 25 dogs with an average age of 7.8 ± 0.2 months and from mixed breeds were used. Following implant insertion, dogs entered estrus (EST; n = 6); dogs without estrus (N-EST; n = 10) comprised the experimental groups. Nine dogs with placebo implants served as a control (CONT). Ovarian and uterine tissues were investigated for expression of GnRH1, GnRHR, KISS1, and KISS1R/GPR54 mRNA and protein by using IHC and RT-qPCR. In the uterus, expression of GnRH1 significantly decreased in response to deslorelin treatment in the N-EST, compared with the control group. Compared with CONT, KISS1R expression in ovarian samples was significantly lower in the EST group. Uterine protein expression of GnRH1 appeared weaker in N-EST than in CONT. While GnRH1-system members and KISS1 protein were localized in the follicles at various stages and stroma, no or only weak signals were detected for KISS1R in the ovarian samples. Deslorelin-mediated induction of puberty by changing the expression of some of the GnRH and KISS1-system members seems to have an effect on ovarian and uterine functionality. Deslorelin implants can, therefore, not be considered a valuable alternative to induce fertile estrus in late-prepubertal bitches. However, further studies with a larger number of animals are needed to clarify the effect of deslorelin-mediated induction of puberty.

## Simple Summary

The GnRH agonist deslorelin may act directly at the ovarian level as well as at the pituitary level in dogs and may directly affect steroidogenesis through ovarian GnRH and KISS receptors.

## 1. Introduction

The physiological initiation of puberty and its regulation is still poorly understood. Few studies address the endocrine changes during puberty of female dogs [1,2,3,4] and the morphological development of the genital tract [5,6,7,8], and only a few of these address estrogen, progesterone [7], and follicle-stimulating hormone (FSH) [5] receptor expression. Many details are still derived from other mammals. However, in prepubertal beagle dogs, at the age of 4 months, chronic infusion of a GnRH agonist prevented the increase in gonadotropins, which stayed at the level of anestrus bitches [3]. This pioneer finding initiated the development of long-term gonadotropin-releasing hormone (GnRH) agonists for the delay of estrus and puberty in dogs [9,10,11,12,13,14,15].

GnRH is a neuroendocrine decapeptide crucial for the onset of puberty and maintenance of reproductive function in mammals [16,17]. During pubescence, the strong central inhibition on gene activation decreases, and increasing amounts of GnRH traverse the portal system to reach the anterior pituitary gland, where it selectively binds to the GnRH receptor (GnRHR) located on gonadotropic cells. Activation of the GnRHR initiates intracellular signaling cascades, subsequently promoting the biosynthesis and secretion of gonadotropins, namely LH and FSH [18,19,20]. Kisspeptins, a family of RF-amide peptides varying in length from 10 to 54 amino acids, play a crucial role in this essential step of sexual ripening [21]. Encoded by the KISS1 gene, kisspeptins exert potent stimulatory effects on gonadotropin release across diverse mammalian species, including humans [22], monkeys [23], sheep [24], rats [25,26], mice [27], and dogs [28].

Kisspeptin is responsible for the regulation of pulsatile GnRH secretion and the onset of puberty [29]. The responsiveness of GnRH neurons to kisspeptin undergoes developmental regulation, with the proportion of responsive GnRH neurons increasing from 25% in prepubertal animals to more than 90% in adults. This increase in GnRH responsiveness during the pubertal phase is attributable to an upsurge in KISS1 gene expression [30].

Shortly before puberty, the slow increase in kisspeptin and GnRH pulses secreted from the hypothalamus is accompanied by increased sensitivity of their receptors and desensitization of steroid hormone receptors in the hypothalamus. During this period, upregulation of estrogen receptors in the central nervous system occurs, as well as receptor-level upregulation of other organs that become increasingly sensitive to estrogen [3]. In beagles, at an average age of 4 months, increasing gonadotropin secretion finally stimulates the ovary to generate gametes, peptides, and steroid hormones in the pubescent animal [3]. The intricate interplay of positive and negative feedback mechanisms in the hypothalamus and pituitary gland then orchestrates the postpubertal regulation of the reproductive hormone axis [31]. Kisspeptin cells play a key role in the steroid negative-feedback control of GnRH neurons, and there is a high degree of co-receptor localization for gonadal sex steroids in these cells. Most kisspeptin cells co-localize the estrogen receptor (ER-alpha—responsible for the negative-feedback actions of estradiol on GnRH secretion), the progesterone receptor, and the androgen receptor [31,32,33].

KISS1 furthermore acts on peripheral reproductive tissues, and the expression of KISS1 and KISS1R genes in canine follicles (granulosa, theca, oocyte), uterus, and trophoblast cells has been determined [34,35]; similarly, in livestock [36]. KISS1 and KISS1R proteins were found in the anteroventral periventricular nucleus and the arcuate nucleus. KISS1 appears to have autocrine and paracrine effects in follicle and oocyte maturation, trophoblast development, and implantation and placentation in mammals [36].

GnRH analogs, which are synthetically produced GnRH, are produced to interact with the GnRH receptor and act on the release of the pituitary gonadotropins FSH and LH. The use of long-acting GnRH agonists for the delay of puberty, duration of action, and flare-up in female dogs is a current research topic; however, there are few scientific studies investigating their clinical and histopathologic/molecular effects on ovarian/uterine function of pubescent dogs [10,12,13,14,15]. Previous studies on the expression of GnRH and KISS1 and their receptors in the uterus and ovaries after deslorelin implant insertion were conducted with very young prepubertal animals at the age of 4 months [13,14]. The assumption was that in these animals, no flare-up should occur. But even at such an early age, results varied considerably, pointing towards different degrees of ripening of the HPG axis and peripheral receptor expression and/or additional factors.

Deslorelin, employed for the long-term suppression of puberty, does not have adverse effects on subsequent reproductive activity and ovarian functionality in dogs [13]; however, there is still insufficient research on the stimulation of puberty and local receptor expression in reproductive organs. Therefore, this study aims to address this gap by investigating the expression and localization of GnRHR and KISS1R in genital tissues following the application of deslorelin in still prepubertal dogs at the age of 7 months.

## 2. Materials and Methods

### 2.1. Animals

In the present study, ovarian and uterine tissues of a total of 25 clinically healthy female dogs were utilized. The tissue samples were derived from our previous study and also utilized in this work [15]. Briefly, dogs of mixed breeds with an average age of 8 months (7.8 ± 0.2), and an average body weight of 21.8 ± 0.7 kg, were brought to Kafkas University Veterinary Faculty Education, Practice and Research Hospital by their owners for ovariohysterectomy (OHE). All dogs were still in the prepubertal period, as was confirmed by anamnesis and clinical/laboratory findings (vaginal inspection, cytology, serum-P4 and -E2 measurements, X-rays of epiphyseal plates). Animal owners were informed about the study before the operation, and their consent was obtained.

### 2.2. Experimental Design and Tissue Collection

**Figure 1:**
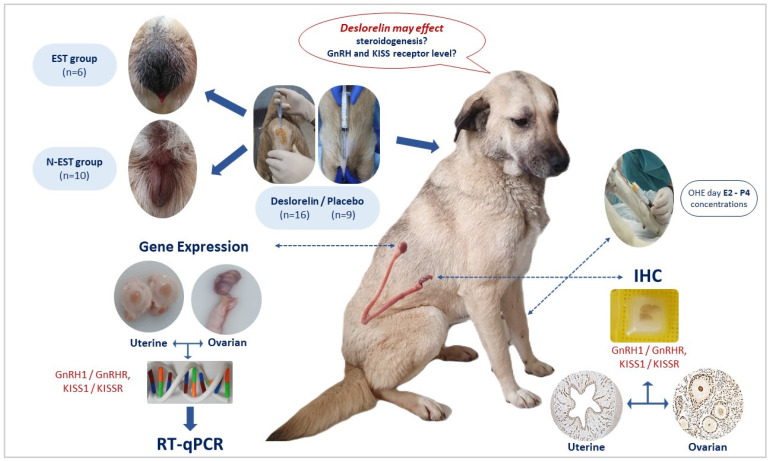
Study design.

Following the clinical examination, a subcutaneous implant containing deslorelin acetate (Suprelorin^®^, 4.7 mg, Virbac, France) was administered into the interscapular region of sixteen late prepubertal dogs [15]. In order to determine estrus, daily clinical observations were performed (vulvar appearance/swelling and bloody vaginal discharge, behavioral changes), and sampling (vaginal cytology and serum estradiol 17β (E2) and progesterone (P4) concentrations) was conducted at 2-day intervals. Dogs exhibiting clinical proestrus/estrus comprised the estrus group (EST, n = 6), while dogs without clinical proestrus/estrus constituted the non-estrus group (N-EST, n = 10). The cycle stages were defined as described by Karadağ et al. [15]. The control group consisted of prepubertal dogs that received placebo implants (CONT, n = 9), with 1 mL of sterile NaCl injected subcutaneously into the interscapular region using an implant applicator. They were otherwise treated like the experimental dogs. Between days 30 and 45 after implant application, ovariohysterectomy (OHE) was performed in all dogs, and tissues were subsequently collected as described below (Figure 1). All operations were performed under general anesthesia. Atropine sulfate (Atropine^®^, 2 mg/mL concentration; Vetas, Türkiye) was administered at a dose of 0.05 mg/kg, SC before anesthesia to support the induction of anesthesia. Premedication was administered with xylazine HCl (Rompun^®^ 23.32 mg/mL; Bayer, Türkiye) at a dose of 2.35 mg/kg, IM. Fifteen minutes after sedation was achieved, ketamine HCl (Ketasol^®^, 100 mg/mL; Interhas, Türkiye) was administered at a dose of 10 mg/kg IM. Anesthesia was then maintained with IV ketamine HCl injection. All animals received prophylactic antibiotics (cefazolin sodium; 30 mg/kg/day, IM, Iespor^®^ 500 mg vial; I.E., Ulugay, Türkiye), and analgesic (meloxicam; 0.2 mg/kg, SC, Maxicam^®^ 5 mg/mL; Sanovel, Türkiye) treatment for 3 days after OHE.

### 2.3. Blood Sampling and Hormone Measurements

Blood samples were collected from all dogs before OHE via the Vena cephalica antebrachii, either into serum tubes without anticoagulant or plasma tubes (BD Vacutainer^®^, Franklin Lakes, NJ, USA) containing EDTA anticoagulant. The samples were then centrifuged (Nüve NF400, Ankara, Türkiye) at 3500 rpm for 10 min, and serum samples were separated.

The samples were stored at −20 °C until hormone measurements were performed using validated assays. Estradiol (E2, pg/mL) and progesterone (P4, ng/mL) concentrations were measured by direct chemiluminescence (CLIA) method using Siemens (Siemens^®^, Tarrytown, New York, NY, USA) fully automatic hormone analyzer and ADVIA Centaur test analysis kits. The ADVIA Centaur test is a measurement technique with a sensitivity of 100% and a specificity of 95.5% [15].

### 2.4. Tissue Processing

Following sectioning based on their anatomical structure, tissues designated for Real-Time qPCR (RT-qPCR) analysis were promptly frozen in liquid nitrogen and stored at −80 °C. The remaining portions of the ovarian and uterine tissues underwent a fixation protocol for immunohistochemical analysis. These tissues were initially placed in a 4% buffered formalin solution at +4 °C for 24 h and subsequently washed with phosphate-buffered saline (PBS) for 7 days. Post-washing, the tissues were dehydrated in a graded ethanol series and embedded in paraffin, as described previously [37].

### 2.5. Real-Time qPCR Analysis

Total RNA extraction was performed using Trizol reagent (TRIzol^®^, Invitrogen, Carlsbad, CA, USA) following the manufacturer’s protocol. The concentrations of RNAs were determined using a NanoDrop™ 2000c spectrophotometer (Thermo Fisher Scientific, Wilmington, DE, USA). In the next step, RNA concentrations of uterine and ovarian tissues were adjusted to 1000 ng. Reverse transcriptase was performed with Oligo (dT) and Random Hexamer primers. Reverse transcriptase enzyme (Sensifat cDNA Kit) (Bioline; Meridian Biosince, Memphis, TN, USA) was used for cDNA synthesis. Primer designs of these kits were made as exon–exon junctions. In case of any DNA contamination, primers do not bind to DNA. Therefore, DNAse treatment was not necessary to prevent contamination. cDNA component volumes were the following: cDNA Buffer 5X 2 µL, RT Enzyme (Sensifat cDNA Kit) 0.5 µL, and RNA + ddH2O = 7.5 µL; the total volume was adjusted to 10 µL. The ABI Veriti 96 (Applied Biosystems. Veriti™ 96-Well Fast Thermal Cycler) facilitated the reverse transcription process with the following temperature steps: 25 °C for 10 min for primer binding, 42 °C for 15 minutes for reverse transcription, and 85 °C for 5 min for enzyme inactivation. Following this, the enzyme was further inactivated at 85 °C for 5 min, and the cDNA products were stored at −20 °C for subsequent use in PCR analysis.

In RT-qPCR analyses, the expression levels of 4 target genes (GnRH1, GnRHR, KISS1, and KISS1R) were analyzed in uterine and ovarian tissues. Three different reference genes (EIF4H, KDM4A, PTK2) were used as an internal control (Table 1). Primer design for each gene was performed using Primer Express Software version 3.0 (Applied Biosystems by Thermo Fischer). After primer design, synthesis of target and reference genes was performed at Biomers company (Ulm, Germany).

The mRNA transcript levels of each gene of interest were determined using the SensiFAST^™^ SYBR^®^ RT-qPCR Kit (SensiFAST SYBR No-ROX Kit, (Meridian Bioscience, Cincinnati, OH, USA)). The mRNA expression levels of each gene of interest were determined following the Bio-RAD protocol using the CFX^®^96 Real-Time system. The 10 μL reaction mixture consisted of 5 μl Master mix (SensiFAST SYBR^®^ No-ROX Mix (Meridian Bioscience)), 2.5 μL mRNA MixB (Primer F and R), and 2.5 μL cDNA sample corresponding to 1000 ng total RNA. As negative controls (NTC), 2.5 μL sterile distilled water (PCR Water, DNase/RNase free, Bioline; Meridian) was used instead of cDNA to screen for DNA contamination. All samples were performed in 96-well plates and duplicates. Amplification conditions were as follows: Initial denaturation at 95 °C for 1 min, followed by 40 cycles of GnRH1 at 61 °C, GnRHR at 57 °C, KISS1 at 65 °C, KISS1R at 59 °C for 30 s, and 1 cycle of Melting Curve analysis at 95°C for 10 s, 70 °C for 5 s, 95 °C for 5 s. Upon completion of the procedure, the assessment of target genes (GnRH1, GnRHR, KISS1, KISS1R) was carried out using the comparative CT method (ΔΔCT method or Livak method). This method involves the calculation of the ΔΔCt value using an arithmetic formula, comparing the CT value of the target gene with the result of the endogenous reference gene, ultimately expressed as 2^-(ΔΔCt).

### 2.6. Immunohistochemistry

In order to determine the immunolocalization of GnRH1, GnRHR, KISS1, and KISS1R in ovarian and uterine tissues, 2 µm thick sections were taken with a microtome device Leica Rm 2125Rt (Leica Biosystems, Nussloch, GmbH, Germany) from the paraffin blocks. The sections were then mounted on HistoBond^®^ slides (Marienfeld, Lauda-Königshofen, Germany) and kept in the oven at 37 °C for 24 h. The streptavidin–biotin immunoperoxidase complex technique was used for the detection of GnRH1, GnRHR, KISS1, and KISS1R. Sections were deparaffinized in xylene, and slides were rehydrated using a graded sequence of ethanol. Epitope/antigen recovery of the deparaffinized slides was performed by heating in 10 mM citrate buffer (pH 6.0, Citrate, Sodium citrate (tri-sodium citrate dihydrate)) at 600 W in a microwave oven for 15 min. Then, the sections were allowed to cool at room temperature for 20 min and then washed with PBS. Endogenous peroxidase activity (to prevent false positive detection and high background staining) was blocked with 3% hydrogen peroxide in absolute methanol for 20 min at room temperature, and then slides were washed with PBS. To prevent non-specific binding of antibodies, 1 drop of blocking solution (Ultra V Block, Thermo Fisher Scientific, LabVision Corporation, Fremont, CA, USA) was added to the sections, and they were incubated for 5 minutes at RT. Tissue sections were then incubated with the primary antibodies (Table 2) overnight at 4 °C. All antibodies were diluted in an antibody diluent solution (Antibody Diluent OP Quanta, Thermo Fisher Scientific, LabVision Corporation, Fremont, CA, USA). For negative controls, samples incubated with nonimmunized IgGs (isotype controls) of the same species and at the same protein concentration as the primary antibody, as well as without the primary antibodies (negative control), were used. The next day, after rinsing the samples with PBS, the slides were incubated with secondary antibodies, i.e., Biotinylated Goat Anti-Rabbit Secondary (Thermo Fisher Scientific, LabVision Corporation, Fremont, CA, USA) for 20 min. After rinsing the slides with PBS, signal intensity was increased by incubating the tissue samples with streptavidin peroxidase (Thermo Fisher Scientific, TS-125-HR) for 20 min at room temperature. Peroxidase activity was obtained by waiting for 10 min using the DAB plus substrate system (Dako, Agilent Technologies, Santa Clara, CA, USA). Slides were counterstained with Gill’s hematoxylin for 5 min and rinsed under tap water. Finally, slides were mounted in Entellan (Entellan^®^, Merck, 1079610500, Darmstadt, Germany).

Immunostaining in the slides was examined using a light microscope (Olympus BX51), and the results were systematically tabulated based on the localization of stained cells within the tissues. Both descriptive and quantitative assessments of brown precipitation and immunostaining were conducted by the same investigator, considering the expression intensity. The evaluation criteria were as follows: no immunoreactivity observed in cells at high magnification (bar: 20 μm-X40) was classified as a negative (−) reaction. Immunoreactivity observed solely in cells at high magnification (bar: 20 μm-X40) was categorized as a weak reaction (+/−). Immunoreactivity in cells at low magnification (bar: 50/100 μm-X10/X20) was considered a strong reaction (+). Clear observation of immunoreactivity in cells at low magnification (bar: 50/100 μm-X10/X20) was designated as a very strong reaction (++).

### 2.7. Statistical Analysis

Statistical analyses were performed using the IBM SPSS 22 package program (SPSS^®^, IL, Chicago, USA). Results are presented as mean ± standard error of the mean (SEM). The normal distribution of steroid hormone values was assessed using the Shapiro–Wilk analysis. Since serum E2 concentration comparison of groups showed normal distribution using one-way analysis of variance (One-Way ANOVA), and P4 concentrations did not show normal distribution, the Kruskal–Wallis analysis was used. In the determination of gene expression levels, one-way analysis of variance (One-Way ANOVA) was used for comparisons since the group analyses provided normal distribution. A post-hoc multiple comparison test, Tukey, was used to determine the differences between the groups. Differences were considered statistically significant if *p* < 0.05.

## 3. Results

In this study, 6 (37.5%) of 16 dogs showed signs of proestrus/estrus within 8.6 ± 0.6 days after implant application, while in 10 dogs, no clinical signs of proestrus/estrus were observed within this period (62.5%). For time to proestrus/estrus and diestrus and duration of proestrus/estrus in individual dogs of the EST group after implant insertion, see Karadag et al. 2023 [15], Table 2. None of the CONT dogs showed clinical or cytological signs of beginning proestrus/estrus.

### 3.1. Serum Estradiol and Progesterone Concentration

**Figure 2:**
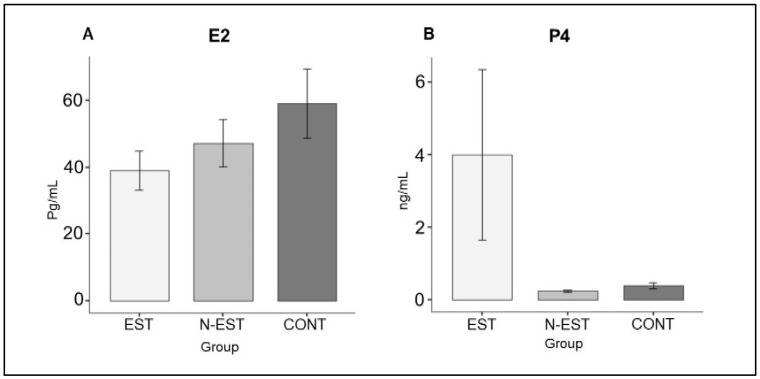
Mean serum E2 (**A**) and P4 (**B**) concentrations of the groups on the day of ovariohysterectomy. (EST: Estrus, N-EST: Non-Estrus, CONT: Control, the sensitivity of the measurements is 100%, and the specificity is 95.5%).

The course of the average serum concentrations of E2 and P4 have been published previously by Karadağ et al. [15]. Within the frame of this study, only the values on the day of OHE were of interest; at this time point, only 4/6 dogs in the EST group and none of the other groups had P4 values >2 ng/mL, indicating ovulation. However, the average serum steroid hormone values (E2 and P4) did not differ significantly between groups on the day of ovariohysterectomy (*p* > 0.05, Figure 2).

### 3.2. Gene Expression of GnRH1, GnRHR, KISS1, and KISS1R in the Uterus and Ovary

**Figure 3:**
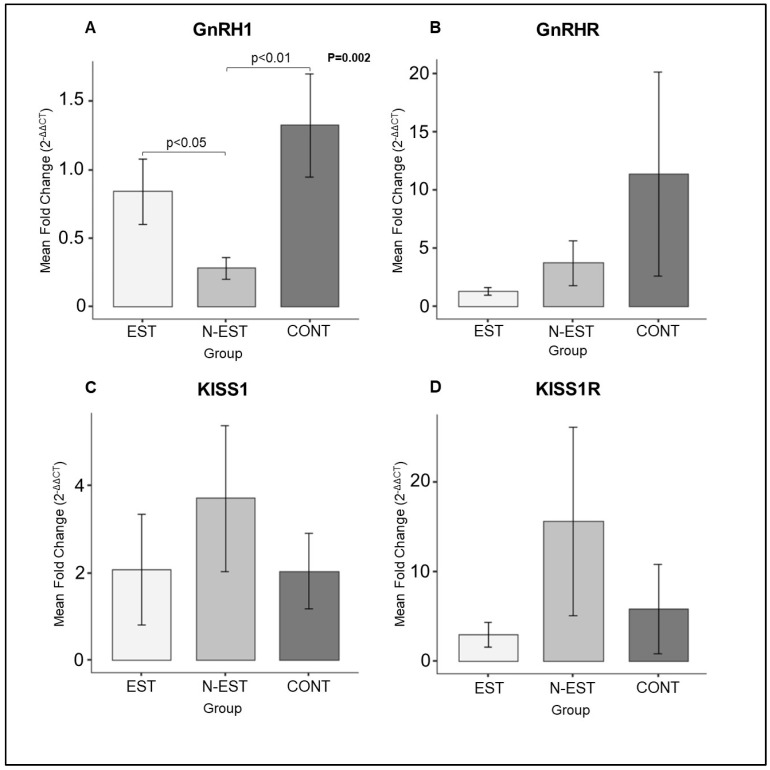
Expression of GnRH1 (**A**), GnRHR (**B**), KISS1 (**C**), and KISS1R (**D**) in uterine tissue. (EST: Estrus, N-EST: Non-Estrus, CONT: Control). All numerical data are presented as mean ± SEM. *p* values < 0.05 were considered significant and indicated. For comparison between groups, the parametric one-way ANOVA was followed by the Tukey multiple comparison post-hoc test.

Expression of GnRH1, KISS1, and their receptors GnRHR and KISS1R was detectable in all uterine and ovarian samples in each group investigated in the present study. As for the uterine samples, expression of GnRH1 mRNA was affected by the deslorelin treatment. Thus, whereas it was unaffected in EST, it decreased significantly in N-EST compared with the CONT animals. However, the abundance of KISS1, GnRHR, and KISS1R transcripts did not differ significantly in each group investigated (Figure 3).

**Figure 4:**
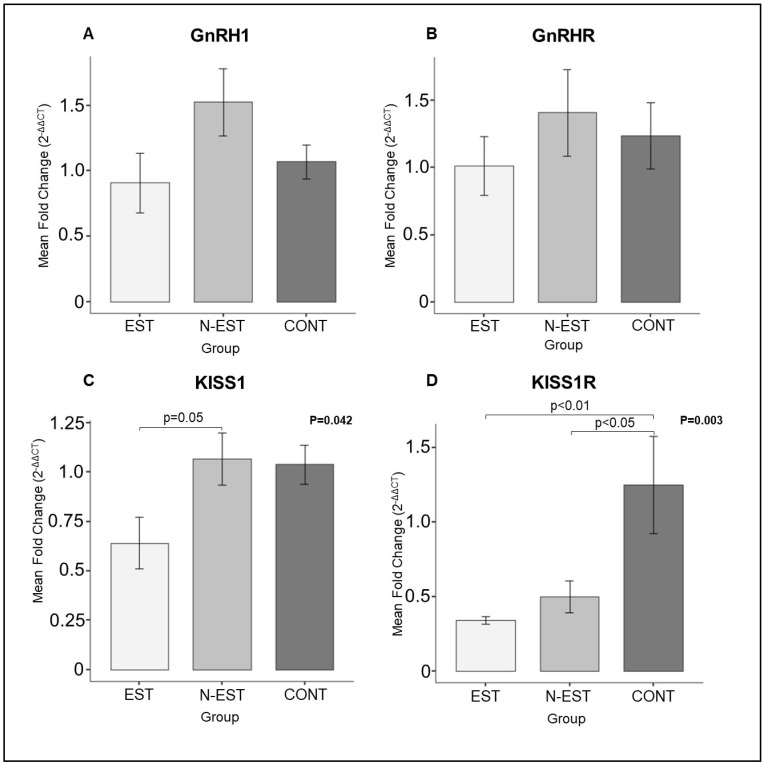
Expression of GnRH1 (**A**), GnRHR (**B**), KISS1 (**C**), and KISS1R (**D**) in ovarian tissue. (EST: Estrus, N-EST: Non-Estrus, CONT: Control). All numerical data are presented as mean ± SEM. *p* values < 0.05 were considered significant and indicated. For comparison between groups, the parametric one-way ANOVA was followed by the Tukey multiple comparison post-hoc test.

In the ovarian samples, the expression of GnRH and GnRHR mRNA did not change significantly in all groups investigated, whereas the expression of KISS1 and KISS1R was significantly affected by deslorelin treatment. Accordingly, the expression of KISS1 mRNA was downregulated in the EST group compared with the N-EST animals. Similarly, in EST animals, KISS1R mRNA decreased significantly compared with the control group (Figure 4).

### 3.3. Localization of GnRH1, GnRHR, KISS1, and KISS1R in the Uterus and Ovary

**Figure 5:**
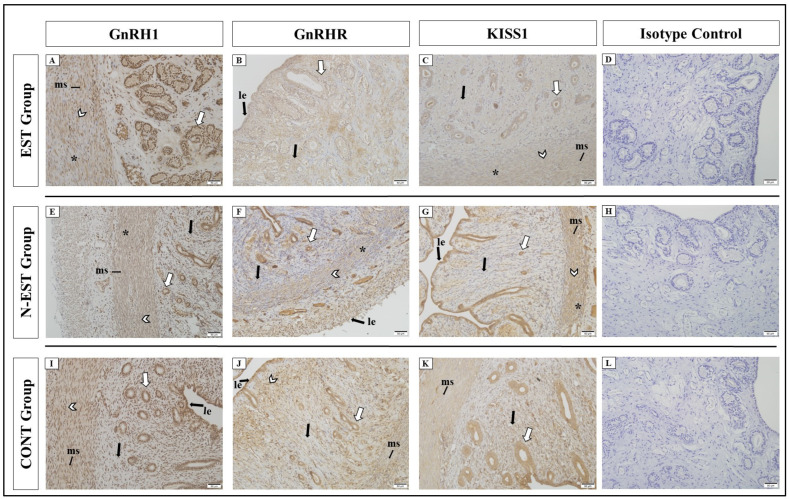
Immunolocalization of GnRH1, GnRHR, and KISS1 in uterine tissue. Immunoreactivity of GnRH1 in different areas of the uterus by groups; EST (**A**), N-EST (**E**), CONT (**I**), (bar: 50 μm-X20). Immunoreactivity of GnRHR in different areas of the uterus by groups: EST (**B**), N-EST (**F**), CONT (**J**), (bar: 50 μm-X20). Immunoreactivity of KISS1 in different areas of the uterus by groups: EST (**C**), N-EST (**G**), CONT (**K**), (bar: 50 μm-X20). No reaction was observed in the isotype controls (**D**,**H**,**L**, bar: 50 μm-20x) (ms: myometrial stroma, le: luminal epithelial cells, black thick arrow: endometrial stroma, arrowhead: myocytes, white arrow: endometrial glands, *: interstitial cells).

In all groups, strong GnRH1 immunoreactivity was detected in luminal epithelial cells, endometrial glands, and superficial and deep uterine glands of the uterus, except the endometrial stroma. A weaker reaction was seen in the myometrium (myocytes, interstitial cells, and myometrial stroma). Similar to GnRH1, GnRHR also showed positive immunoreactivity in immunohistochemical sections in all groups (Figure 5).

All groups showed different degrees of positive immunoreactivity for KISS1 in luminal epithelial cells, endometrial glands, superficial and deep uterine glands, myocytes, interstitial cells, and endometrial stroma. While similar positive immunoreactivity was observed in cellular localizations of the uterus between groups, in the N-EST group, strong immunoreactivity was seen in luminal epithelial cells and superficial uterine glands. For KISS1R, overall negative and weak positive immunoreactivity was observed in cellular localizations of the uterus. Very weak and weak immunoreactivity was observed in endometrial glands, superficial and deep uterine glands, endometrial stroma, and interstitial cells, while KISS1R showed no immunoreactivity in luminal epithelial cells, myocytes, and endometrial stroma in some groups (Figure 5, Table 3).

**Figure 6:**
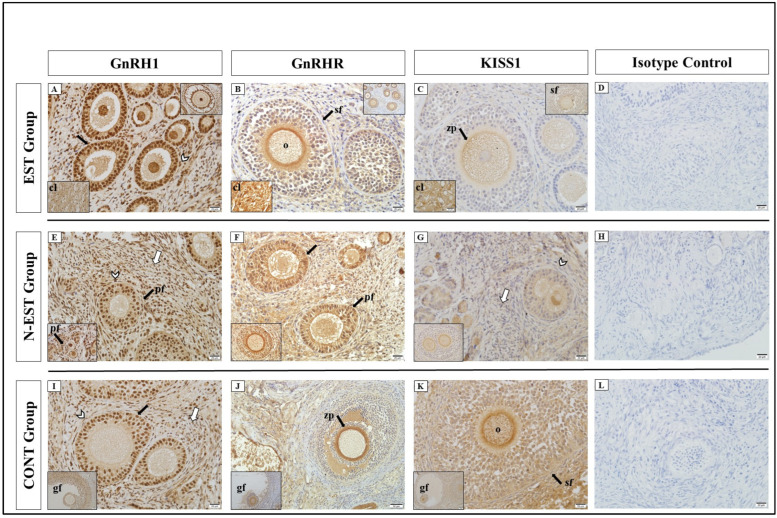
Immunolocalization of GnRH1, GnRHR, and KISS1 in ovarian tissue. Immunoreactivity of GnRH1 in different areas of the ovary by groups; EST (**A**), N-EST (**E**), CONT (**I**), (bar: 20 μm-X40). Immunoreactivity of GnRHR in different areas of the ovary by groups: EST (**B**), N-EST (**F**), CONT (**J**), (bar: 20/50 μm-X20/40). Immunoreactivity of KISS1 in different areas of the ovary by groups: EST (**C**), N-EST (**G**), CONT (**K**), (bar: 20 μm-X40). No reaction was observed in the isotype controls (**D**,**H**,**L,** bar: bar: 20/50 μm-X20/40). (cl: corpus luteum, gf: Graafian follicle, sf: secondary follicle, pf: primary follicle, zp: zona pellucida, o: oocyt, black arrow: granulosa cell, arrowhead: theca cell, white arrow: endocrine interstitial cell).

GnRH1 showed positive immunoreactivity in cellular localizations of the ovary in all groups. Similar to GnRH1, positive immunoreactivity for GnRHR was determined at different degrees (strong, very strong) in the groups. In all groups, very strong GnRH1 immunoreactivity was detected in primary, secondary, and graafian follicles, oocytes, granulosa, and theca cells, and strong immunoreactivity in the zona pellucida and endocrine interstitial cells. For KISS1, the weakest immunoreactivity was seen in theca cells in all groups. In all groups, KISS1R showed negative and weak positive immunoreactivity in all cellular localizations of the ovary in all groups. The weakest immunoreactivity was seen in theca cells and endocrine interstitial cells (Figure 6, Table 4).

## 4. Discussion

### 4.1. Steroid Hormone Concentrations

In various studies involving adult dogs, both stimulation and suppression of estrus have been successfully achieved through the administration of GnRH agonists. The effect was mainly dependent on the cycle stage at the time of application but also, sometimes, unknown individual factors. In prepubertal dogs, the mechanism of prevention/delay of initiation of the hypothalamus–pituitary–gonadal axis (HPG) by deslorelin is not yet fully understood. Most probably, the individual grade of immaturity of GnRH neurons, the kisspeptin/GnRH pulse-producing neuronal circuit, and further unknown control mechanisms regulating the secretory activity of neurons [38] result in distinct clinical findings. Clinical studies have unveiled variable suppression durations and inconsistent flare-up findings even when application took place as early as 3–4 months of age [9,10,12,13,39]; however, similarly, when implants were inserted at the age of 6–8 months [9,15,40]. These results may suggest that the effects of prepubertal administration of the GnRH agonist deslorelin on the HPG axis are not limited to central GnRH release alone. Other factors, such as peripheral expression of GnRH, kisspeptin (KISS), and their receptors, which are essential for reaching puberty, should also be investigated. Therefore, in this study, the presence and localization of GnRH1 and KISS1 and their receptors in ovarian/uterine tissues after insertion of deslorelin in late prepubertal dogs were investigated. The most critical point of the studies on the prepubertal period is to determine with clinical and hormonal analyses that the animals are actually prepubertal [41]. The prepubertal period was confirmed by clinical reproductive examinations, vaginal cytology, determination of E2/P4 levels, and X-ray control of epiphysial closure in all female dogs used in this study.

Studies on the application of deslorelin in prepubertal dogs at the age of 3–4 months revealed a variable response in serum E2 concentrations [12,13,14]. Some animals exhibited an increase followed by a rapid decrease below 18 pmol/L, maintaining a low level throughout the treatment period without showing clinical signs of proestrus. Only 4/9 animals entered proestrus, and only two out of these ovulated, as indicated by the P4 values. Mean E2 and P4 concentrations at the time of tissue removal in control and deslorelin-treated bitches 30–45 days after they had entered proestrus did not differ significantly between deslorelin-treated bitches and controls [12,13,14]. The authors explained the possible reasons for this with individual differences in drug absorption and/or metabolism between dogs. Differences in individual deslorelin concentrations during the first four weeks in dogs administered different doses of deslorelin acetate support this view [12]. In this study, which solely included late prepubertal dogs at the age of 7 months, the serum E2 values in the EST group were lowest at 30–45 days after implant insertion, indicating post-flare-up decrease, whereas in the CONT group, the higher average value may indicate approaching proestrus. Average progesterone values were relatively high in the EST group since 4/6 dogs had ovulated, others not; the latter may be related to inadequate release of neuropeptides, hormones, and mediators effective in the stimulation of puberty rather than the effect of deslorelin. Anovulatory cycles rather frequently occur in young bitches during their first cycle; however, this occurred both after deslorelin administration at the age of 4 and 7 months. Finally, these results so far indicate that the effect of deslorelin on prepubertal dogs cannot be completely foreseen; clinical parameters E2 and P4 values do not sufficiently indicate how the individual HPG axis will react, not even in 3–4-month-old animals; on the other hand, insertion of deslorelin in older dogs does not necessarily result in a flare-up. This rectifies the search for other indicators of approaching puberty.

### 4.2. Expression of GnRH1, GnRHR, KISS1, and KISS1R Genes and Proteins in the Ovary

#### 4.2.1. KISS1 and KISS1R

The findings from our study show the presence of KISS1 and KISS1R proteins and mRNA in the ovarian tissue of dogs before and after puberty. In the ovary, the presence of KISS1 and its receptor is thought to fulfill multiple functions during the female cycle. Its role in follicular development is recognized by the fact that in rats, the mRNA of KISS1 in the ovary gradually increases from birth to puberty [42]. High expression of KISS1 and KISS1R in the ovaries of different mammals, such as rodents, hamsters, and humans, during the pubertal period, has been previously reported [43,44,45,46,47]. A recent study showed that kisspeptin controls ovulation and follicular dynamics in mice, and its deficiency is one of the main factors responsible for ovarian aging in women [48,49]. Gaytan et al. [48] reported in their study in mice that kisspeptin receptor deficiency causes early ovulatory failure; KISS1R hypomorph mice showed an early decline in ovulation rate, followed by progressive loss of antral follicles, oocyte loss, and a decrease in all categories of preantral follicles. They also reported that the failure of follicular development and ovulation due to KISS1R deficiency cannot be completely corrected by (prolonged) gonadotropin replacement. These findings point to a direct active role of kisspeptin signaling in the ovary.

The similarity between the groups in the distribution of KISS1 and KISS1R in the ovary at 30–45 days after implant insertion and irrespective of a flare-up and whether dogs had ovulated or not, indicates the physiological site of kisspeptin signaling, especially in the oocyte, in the control of ovulation and follicle survival, and independent of the agonistic effect of deslorelin; the latter might mainly change the degree of expression in case of a flare-up by promoting physiological downregulation towards the luteal phase. The latter was supposed since the ovarian KISS1 and KISS1R expression in the EST group was significantly lower than in the other groups, probably suggesting that KISS1 had exerted its local effect earlier, whereas, at the time of tissue collection, deslorelin or another mechanism had already promoted local downregulation of these genes. However, this hypothesis has to be proven.

Ruohonen et al. 2022 [49] proposed that the expression of KISS1 and KISS1R, along with the presence of KISS1 and KISS1R proteins in follicles (granulosa cells, theca cells, oocytes) and corpus lutea in mice, exerts significant autocrine and paracrine effects on local KISS1, influencing ovarian function. In a study by Cielesh et al. 2017 [34] on dogs, the immunohistochemical localization of KISS1 and its receptor in ovaries was investigated. The study involved prepubertal (n = 5), anoestrus (n = 5), and cyclic animals (proestrus, estrus, and dioestrus; n = 14), ranging from 10 weeks to 10 years of age. Unfortunately, the average age of the prepubertal group was not given; puberty was excluded post-operation by examination of the uterus and the ovaries. Expression of KISS1 was found in primordial follicles of one prepubertal bitch, and expression of KISS1R in primordial follicles, oocytes, and granulosa cells of all bitches. However, no positive reaction was detected in theca cells. In postpubertal dogs with corpora lutea, staining of KISS1 and KISS1R was inconsistently detected in granulosa cells, oocytes, and corpora lutea [34]. In our study, KISS1 showed weak immunoreactivity in theca cells in all groups, also in those that had not entered proestrus at the time of OHE, due to either a delay of puberty or naturally late occurrence of puberty (N-EST and CONT); KISS1R showed negative or weak immunoreactivity in all cellular localizations of the ovary in all groups with weakest immunoreactivity in theca cells and endocrine interstitial cells. The discrepancy in theca cell expression of KISS1 and KISS1R may be due to different stages of ovarian development in dogs of both studies. The role of kisspeptins in oocyte development and survival may increase as puberty approaches. KISS1R expression in the mid-luteal corpus luteum of deslorelin-treated early prepubertal dogs did not differ from controls. KISS1 protein expression was low in both groups [13]. Similarly, in this study on late prepubertal dogs, protein expression of both KISS1 and KISS1R was weak but detectable in the corpus luteum of the EST group during the first mid-luteal phase. Thus, in dogs, kisspeptin, produced in and/or acting on granulosa cells of the ovary, may be important for progesterone production/secretion. In a recent study, a bolus injection of Kiss-10 in adult anestrus beagle dogs did not induce estrus but seemed to prevent a preovulatory increase in progesterone [50]. It remains to be proven whether this was caused by local KISS1R downregulation.

#### 4.2.2. GnRH and GnRHR

In the present study using late prepubertal dogs at 30–45 days after implant insertion, the ovarian expression of both GnRH and GnRHR was proven and comparable between groups of mid-luteal postpubertal (EST) and prepubertal dogs (N-EST, CONT), irrespective of deslorelin treatment. Protein expression of GnRH1 and -R was variable; in the case of GnRH1, it was strong/very strong in all structures of the ovary except the Graafian follicles of the treated groups. While the significantly lower levels of KISS1 and KISS1R expression in the EST group may suggest that deslorelin treatment accelerates local downregulation of these genes, this does not seem to apply to ovarian GnRH and its receptor; mRNA expression at day 30–45 after implant insertion was comparable between groups. In parallel with the findings of this study in late prepubertal dogs, Kaya et al. 2017 [13] reported that the relative expression of GnRHR and KISS1 was found to be low in ovarian tissue in both deslorelin-treated and control early prepubertal animals. The GnRH/GnRHR system is supposed to contribute to follicle development and atresia and to luteinization as well as luteolysis, and, in a more autocrine/paracrine way, also in ovarian epithelial cell proliferation of different mammals [51,52,53,54]. These processes are supposed to be still inactive or not fully active, especially in very young prepubescent dogs, even though receptors and hormones are already there. However, after puberty, the role of this system in follicular and corpus luteum steroidogenesis and apoptosis [55,56,57,58,59,60] has been demonstrated in several mammalian species; in the canine species, there is no proof. The present results implicate activities of the GnRH/GnRHR system during canine follicular development before puberty and during the first estrus thereafter, as has been described in humans [61]. Results of this study and earlier findings [13] furthermore point towards a direct endocrine regulatory involvement of GnRH within the canine corpus luteum, as in corpora lutea of dogs during their first estrus, GnRH and –R protein and mRNA expression was detected (EST). Time-dependent relative expression of GnRHR in the corpus luteum of adult dogs throughout diestrus and decreasing relative expression with luteal regression has been reported [13]. The effect of deslorlin on canine ovarian GnRH/GnRHR expression may be doubted since no differences between groups were observed.

However, more studies are necessary to investigate the local effect of GnRH/GnRH1R and GnRH agonists in the canine ovary, especially as in other species; differences at the molecular level between the central and the peripheral system were supposed; different intracellular signaling and post-translational processing are possible [62,63].

### 4.3. Expression of GnRH1, GnRHR, KISS1, and KISS1R Genes and Proteins in the Uterus

#### KISS1 and KISS1R

In the uterus, KISS1 protein expression was found in all uterine cells, whereas KISS1R protein was inconsistently expressed and either weak or absent. KISS1 and KISS1R mRNA expression were comparable between groups, but a tendency for higher values was found in treated dogs that did not enter proestrus (N-EST). During a previous study on deslorelin application in early prepubertal dogs, similar results were found as protein expression of KISS1R and ERα,β was especially high in bitches ovariohysterectomized prepubertally [14], which corresponds to the N-EST group of this study; for KISS1, immunoreactivity scores were highest in the postpubertal controls. Expression of KISS and KISS1R mRNA in the human endometrium has been reported; however, in contrary to the present study, not in the myometrium [45]. In mice, kisspeptin binding to intrauterine KISS1R was shown to affect uterine growth and especially gland function [64] and participates in endometrial homeostasis. A lack of KISS1 and KISS1R in the endometrium can cause abnormal expression of matrix metalloproteinases (MMPs), vascular endothelial growth factor (VEGF), and other molecules, leading to disturbed invasion, migration, and angiogenesis of the endometrium; furthermore, abnormal endometrial hyperplasia, endometriosis, and uterine endometrial carcinoma were related to this abnormality. Kisspeptin thus seems to be an important factor for the maintenance of the normal structure and function of the endometrium, also during pregnancy [65]. It was previously stated that in humans, the expression of KISS1 and KISS1R increases significantly during the late secretory phase, coinciding with ongoing decidualization in the pregnant uterus [66]; KISS1 and its receptor were also detected in the canine pregnant uterus and trophoblast cells [35]. In our study, still prepubertal (N-EST, CONT) and meanwhile, postpubertal bitches (EST) were obviously ovariohysterectomized before this increase. However, uterine expression of KISS1 and KISS1R seems to be higher in prepubertal than postpubertal dogs, is highly variable, and seems not to be affected by the stage of prepubescence at the time of deslorelin application.

In contrast to KISS1 and KISS1R, GnRH1 expression in the uterus was significantly lower in the N-EST group than in the other groups, whereas GnRH1R expression did not differ between groups; however, both GnRH1 and GnRH1R expression was highest in the still prepubertal CONT group. This may suggest that exposure of GnRH neurons to a GnRH agonist for approximately 2–3 weeks was sufficient to initiate downregulation of both GnRH and its receptor in the treatment groups. This is supposed since, in humans, an increase in the endometrial expression of GnRH and –R during the luteal phase was observed, especially in glandular and stromal cells [67]; however, this is not proven in dogs, and the here observed effect might also be related to physiological changes during the mid-luteal phase. The protein expression differed between cells but was, on average, more intense for GnRH1 than for its receptor. In all groups, GnRH1 showed strong positive immunoreactivity in all layers of the uterus except the endometrial stroma in all groups. However, GnRHR immunoreactivity was especially weak in the EST group and in all endometrial layers, which might be due to the flare-up or a local effect of deslorelin. In our study, deslorelin might have overcome the progesterone effect by downregulation of local GnRH- and receptor; however, this remains to be proven.

## 5. Conclusions

Finally, in this study, we found signs that GnRH, KISS, and supposedly also the GnRH agonist deslorelin may have a direct effect on both the ovarian and uterine levels in dogs. All may directly affect steroidogenesis through ovarian GnRH- and KISS- receptors, which could be part of a local autoregulation system, as in some other mammalian species. The significantly decreased KISS1 and KISS1R expression in ovarian tissues of dogs in the EST group following deslorelin application and similar numerical findings in GnRH1/GnRHR expression may indicate that the mutual regulatory mechanism between GnRH and KISS is affected by GnRH agonist applications in the late prepubertal period. Our data furthermore reveal that the molecular responses of deslorelin in female dogs in the late prepubertal period show individual differences, similar to the clinical responses and similar to the effects in early prepubertal dogs. This may be related to the fact that puberty is a complex and multifactorial biological process. More animals and better indicators of real prepubescence, before the “brake” in the central nervous system, allows KISS- and GnRH-pulsatility to increase, would be required to better study the effect of deslorelin in prepubertal dogs. Furthermore, the role of ovarian and uterine GnRH and KISS1 receptors in dogs deserves more research in the future.

## Figures and Tables

**Table 1:** List of primer sequence and accession numbers used for real-time (SYBR No-ROX) qPCR. F; forward primer, R; reverse primer.

Primer Sequence			
Gene	Primer Sequences	Product Length (bp)	Accession Numbers (Canis Lupus Familaris)
**GnRH1**	F: 5′-AGTCTGATTGAAGAGGAA-3′	157	XM_038434631
	R: 5′-AATATGTGAACTTAGCGTAA-3′		
**GnRHR**	F: 5′-ACACGAGTCCTTCATCAG-3′	129	AF206513
	R: 5′-AGTCCAGCACACAGTAAA-3′		
**KISS1**	F: 5′-CCTGGTTTCTTGGCAGCTAATG-3′	81	KJ512885 [13]
	R: 5′-GTCTCCATGGGTGCCACCTT-3′		
**KISS1R**	F: 5′-GGAACTCTCTGGTCCTTT-3′	73	NM_001314087
	R: 5′-TTGGCTATGTAAAAGTTGGT-3′		
**EIF4H**	F: 5′-TAAGGTCTCAGCAATTAC-3′	101	XM_014114129
	R: 5′-AAGTTAAGTATTGGTGTCA-3′		
**KDM4A**	F: 5′-TCACAGAGAAGGAAGTTAAG-3′	82	XM_005629107
	R: 5′-GCAGGCTCAATGTAATCT-3′		
**PTK2**	F: 5′-ACCTGGCTGACTTCAATC-3′	85	XM_005627993
	R: 5′-ATCTTCAACTGTAGCATTCCT-3′		

**Table 2:** List of antibodies and dilutions used in immunohistochemical staining.

Target Protein	Product Reference and Manufacturer	Species/Type	Dilution
**GnRH1**	ORB585773 (Biorbyt, Cambridge, UK)	Rabbit polyclonal	1:100
**GnRHR**	AGR030 (Alomone Labs, Jerusalem, Israel)	Rabbit polyclonal	1:100
**KISS1**	Obtained from Prof.Dr. Alain Caraty (INRA, France)	Rabbit polyclonal	1:100
**KISS1R**	NLS1927 (Novus, Centennial, CO, USA)	Rabbit polyclonal	1:100

**Table 3:** Immunoreactivity of GnRH1, GnRHR, KISS1 and KISS1R at the cellular level in uterine tissue.

	Luminal Epithelial Cells			Endometrial Glands			Uterine Glands						Endometrial Stroma			Myocytes			Interstitial Cells			Myometrial Stroma		
							Superficial			Deep														
**Group**	EST	N-EST	CONT	EST	N-EST	CONT	EST	N-EST	CONT	EST	N-EST	CONT	EST	N-EST	CONT	EST	N-EST	CONT	EST	N-EST	CONT	EST	N-EST	CONT
**Gene**																								
**GnRH1**	++	++	++	++	++	++	++	++	++	++	++	++	+	+	+	+	+	+	+	+	++	+	+	+
**GnRHR**	+	++	++	+	++	++	+	++	++	+	+	++	+	+	+	+	+	+	+	+	+	+	+	+
**KISS1**	+	++	++	+	++	+	+	++	+	+	+	+	+	+	+	+	+	+	+	+	+	+	+	+
**KISS1R**	−	−	−	−/+	−/+	−/+	+	−/+	−/+	+	−/+	−	−	−/+	−	−	−	−	−	−/+	−/+	−	−/+	−

(−): Immunoreactivity none, (+/−): weak, (+): strong, (++): very strong.

**Table 4:** Immunoreactivity of GnRH1, GnRHR, KISS1 and KISS1R at the cellular level in ovarian tissue.

	Primary Follicle			Secondary Follicle			Graafian Follicle			Oocyte			Zona Pellucida			Corpus Luteum			Granulosa Cell			Theca Cell			Endocrine Interstitial Cell		
**Group**	EST	N-EST	CONT	EST	N-EST	CONT	EST	N-EST	CONT	EST	N-EST	CONT	EST	N-EST	CONT	EST	N-EST	CONT	EST	N-EST	CONT	EST	N-EST	CONT	EST	N-EST	CONT
**Gene**																											
**GnRH1**	++	++	++	++	*	++	*	*	+	++	++	++	+	+	+	++	*	*	++	++	++	++	++	++	+	+	+
**GnRHR**	++	++	+	+	*	+	*	*	+	+	++	+	++	++	++	++	*	*	++	++	+	−/+	+	−/+	+	++	+
**KISS1**	+	+	+	+	*	+	*	*	+	+	++	++	+	+	++	+	*	*	+	+	++	−/+	−/+	−/+	+	−/+	+
**KISS1R**	+	−/+	+	+	−/+	−/+	*	*	+	+	+	+	−/+	−/+	+	+	*	*	−/+	−/+	−/+	−/+	−/+	−/+	−/+	−/+	−/+

(*): None, (−): Immunoreactivity none, (+/−): weak, (+): strong, (++): very strong.

## Data Availability

The datasets used during the current study are available from the corresponding author on reasonable request.
